# Utility of EFEMP1 in the Prediction of Oncologic Outcomes of Urothelial Carcinoma

**DOI:** 10.3390/genes12060872

**Published:** 2021-06-06

**Authors:** Tzu-Ju Chen, Ti-Chun Chan, Wan-Shan Li, Chien-Feng Li, Hung-Lung Ke, Yu-Ching Wei, Wen-Jeng Wu, Wei-Ming Li

**Affiliations:** 1 Department of Clinical Pathology, Chi Mei Medical Center, Tainan 710, Taiwan; a108n2@mail.chimei.org.tw (T.-J.C.); a80818@mail.chimei.org.tw (W.-S.L.); cfli@mail.chimei.org.tw (C.-F.L.); 2 Department of Medical Technology, Chung Hwa University of Medical Technology, Tainan 717, Taiwan; 3 Institute of Biomedical Sciences, National Sun Yat-sen University, Kaohsiung 804, Taiwan; 4 Department of Medical Research, Chi Mei Medical Center, Tainan 710, Taiwan; 090807@nhri.edu.tw; 5 National Institute of Cancer Research, National Health Research Institutes, Tainan 704, Taiwan; 6 Institute of Precision Medicine, National Sun Yat-sen University, Kaohsiung 804, Taiwan; 7 Department of Urology, Kaohsiung Medical University Hospital, Kaohsiung 807, Taiwan; hlke@kmu.edu.tw (H.-L.K.); wejewu@kmu.edu.tw (W.-J.W.); 8 Department of Urology, School of Medicine, College of Medicine, Kaohsiung Medical University, Kaohsiung 807, Taiwan; 9 Department of Pathology, School of Medicine, College of Medicine, Kaohsiung Medical University, Kaohsiung 807, Taiwan; ycwei@kmu.edu.tw; 10 Department of Pathology, Kaohsiung Municipal Ta-Tung Hospital, Kaohsiung 801, Taiwan; 11 Center for Liquid Biopsy and Cohort Research, Kaohsiung Medical University, Kaohsiung 807, Taiwan; 12 Department of Urology, Ministry of Health and Welfare, Pingtung Hospital, Pingtung 900, Taiwan

**Keywords:** EFEMP1, bladder cancer, upper tract urothelial carcinoma, survival, prognosis

## Abstract

Urothelial carcinoma (UC) of the upper tract (UTUC) and urinary bladder (UBUC) is a heterogeneous malignancy. Through transcriptomic profiling of the Gene Expression Omnibus UBUC dataset (GSE31684), we discovered that epidermal growth factor-containing fibulin-like extracellularmatrix protein 1 (*EFEMP1*) was the most upregulated gene during metastatic development. EFEMP1 is an important component of basement membranes and acts as an enzyme regulator in extracellular matrix biology. Initially, evaluation of *EFEMP1* mRNA expression in 50 UBUCs showed significantly upregulated levels in high stage UC. We further validated the clinical significance of EFEMP1 in 340 UTUC and 295 UBUC using immunohistochemistry, evaluated by H-score. High EFEMP1 immunoexpression significantly correlated with high pathologic stage, high histological grade, lymph node metastasis, vascular invasion, perineural invasion and high mitosis (all *p* < 0.05). After adjusting for established clinicopathological factors, EFEMP1 expression status retained its prognostic impact on disease-specific survival and metastasis-free survival in UTUC and UBUC (all *p* < 0.01). Furthermore, Ingenuity Pathway Analysis showed that actin cytoskeleton signaling, tumor microenvironment pathway and mitochondrial dysfunction were significantly enriched by *EFEMP1* dysregulation. In conclusion, high EFEMP1 expression was associated with adverse pathological features in UC and independently predicted worse outcomes, suggesting its roles in clinical decision-making and risk stratification.

## 1. Introduction

Urothelial carcinoma (UC) derived from lining of the urinary tract is a common malignant tumor, which mainly affects the elderly and occurs in the upper urinary tract (UT) and urinary bladder (UB) [1,2,3]. Radical nephroureterectomy (RNU) is the standard treatment for UTUC, although kidney-sparing surgery is suggested in the patients with low-risk disease [1]. UBUC can be classified into either muscle-invasive bladder cancer (MIBC) or non-muscle-invasive bladder cancer (NMIBC). Patients with NMIBC should undergo transurethral resection of bladder tumor (TURBT) and subsequent intravesical therapy [2]. Radical cystectomy with perioperative chemotherapy is the standard management for MIBC and high-risk NMIBC [2,3]. UC is a highly heterogeneous malignancy with varied response rates when therapies are administered to unselected patient populations.

Although advances in surgery, chemotherapy protocols, immune checkpoint inhibitors and targeted therapy drugs have improved the clinical outcomes of some patients with UC, the overall prognosis and patient survival remain unsatisfactory [1,2,3]. Although non-muscle-invasive UCs have a relatively high 5-year survival rate (90%), those that progress to muscle invasion have a decreased survival rate (approximately 70% at 5years) [2,3]. Moreover, the 5-year survival rate of patients with metastatic UC is only 5–35% [1,3]. Current clinicopathological features have insufficient accuracy to predict clinical outcomes for each patient [4,5]. Understanding the invasive and metastatic processes of UC is critical to future effective therapy development and disease management.

To identify differentially expressed genes (DEGs) associated with UC progression, we carried out data mining of a transcriptomic dataset. We discovered that epidermal growth factor-containing fibulin-like extracellular matrix protein 1 (*EFEMP1*) was the most upregulated gene, which was significantly related to advanced UC stage and disease metastasis, suggesting its role in cancer progression. EFEMP1, also known as fibulin-3, is a secreted extracellular matrix glycoprotein belonging to the fibulin family [6,7]. It is broadly expressed in the body during development and in adult tissues and is an important component of basement membranes. EFEMP1 also acts as an enzyme regulator in extracellular matrix biology [6,7]. Therefore, abnormalities in its roles may strengthen the significance of the capacity for tumor cell invasion and metastasis in cancer. Recently, a growing number of studies have emphasized the importance of EFEMP1 intumorigenesis [8,9,10,11,12,13,14,15,16,17,18]. Upregulation of EFEMP1has been found in malignant gliomas, osteosarcoma, pancreatic cancer, mesothelioma and leukemia [8,9,10,11,12,13]. However, in the breast, prostate, lung, colorectal and liver cancers, EFEMP1 is downregulated in cancer tissues [14,15,16,17,18]. To date, the possible implication of EFEMP1 in UC has not been well studied. Accordingly, we proposed to assess EFEMP1 expression and its prognostic usefulness in our well-characterized UC cohorts.

## 2. Materials and Methods

### 2.1. Data Mining

To explore the DEGs during UC progression, data mining was initially performed on the Gene Expression Omnibus (GEO) dataset(GSE31684) and analysis of 93 UBUCs using Affymetrix Human Genome U133 Plus 2.0 Array [19]. Raw files were imported into the Nexus Expression 3 software (BioDiscovery, El Segundo, CA, USA) to computerize the expression level as depicted previously [20,21]. We compared tumor stage (MIBC vs. NMIBC) and metastatic events (non-metastasis vs. metastasis) to identify significant DEGs. The top 10 DEGs (log2 ratio > 0.7 and *p* <0.01) were selected for further study.

### 2.2. Collection of Patient Data and Tissues

We enrolled 635 consecutively well-characterized UC patients: 340 UTUC and 295 UBUC between 1998 and 2004. All patients underwent surgery with curative intent. None of the patients received neoadjuvant chemotherapy or radiotherapy before the operation. Histological grading was assigned according to the WHO 2004 grading system, whereas tumor stages were determined based on the 7th edition of the AJCC/UICC TNM staging system. All the samples were verified by two pathologists. We retrospectively reviewed patient characteristics, pathological features and follow-up data. The study was approved by the Institutional Review Board (IRB10302-015).

### 2.3. Quantitative RT-PCR

Total RNA extraction was performed using the Total RNA Purification Kit (GeneMark, Atlanta, GA, USA) according to the manufacturer’s instructions. Purified RNA was subjected to cDNA synthesis using the Maxima First, Strand cDNA Synthesis Kit (Thermo Scientific, Waltham, MA, USA). Subsequently, we measured *EFEMP1* (Hs00244575_m1) mRNA using TaqMan™ Fast Advanced Master Mix (Thermo Scientific, Waltham, MA, USA), Pre-designed TaqMan assay reagents and a StepOne Plus System (Applied Biosystems, Waltham, MA, USA) as previously described [15,16]. The fold of expression of *EFEMP1* relative to adjacent non-tumor urothelium was calculated after normalization to *POLR2A* (Hs01108291_m1) as the internal control.

### 2.4. Immunohistochemistry

All formalin-fixed, paraffin-embedded tissues were cut into 4 μm sections and placed on pre-coated slides. We followed the standard immunohistochemistry protocols, including deparaffinization, rehydration, antigen retrieval and inactivation of endogenous peroxidase, as depicted previously [21,22]. Next, the samples were incubated in the presence of anti- EFEMP1 primary antibody (1:100, LS-C167641, LSBio Inc. Seattle, WA, USA) for 1 h and subsequently incubated with peroxidase-conjugated secondary antibody reagent. The primary antibody was detected using the Dako REALEnVision™ Detection System (Dako Agilent, Santa Clara, CA, USA). The slides were counterstained with hematoxylin. Two independent pathologists assessed the percentage and intensity of positive immunostaining UC cells to generate the H-score, with the following equation:

ΣPi(i +1),

where Pi represents the percentage of stained UC cells for each intensity (0% to 100%) and i is the intensity of stained UC cells(0 to 3+). Immunoreactivity was divided into high and low expression levels using the median H-score.

### 2.5. Ingenuity Pathway Analysis (IPA)

Gene expression levels and clinical data of TCGA-BLCA were downloaded from the cBioPortal (http://cbioportal.org (accessed on 1 December 2020)). We explored the common DEGs between low and high EFEMP1-expressing UCs and uploaded the identified DEGs into Qiagen’s IPA system (http://www.ingenuity.com (accessed on 1 December 2020)) for core analysis. IPA was performed to identify canonical pathways, upstream regulators, diseases and functions and gene networks related to dysregulated *EFEMP1* in UC.

### 2.6. Statistical Analysis

Statistical analysis was performed using SPSS software (IBM, Armonk, NY, USA). We used the Pearson’s chi-square test to assess the association between EFEMP1 expression status and different clinicopathological features. The Kaplan–Meier survival analysis with log-rank test was applied to estimate the effect of EFEMP1 protein level (high vs. low) on patient outcomes, including bladder recurrence-free survival (BRFS), disease-specific survival (DSS) and metastasis-free survival (MFS) measured from curative surgery to the time of bladder tumor recurrence, cancer death and metastatic development. Univariate and multivariate analyses with the Cox proportional hazards model were used to identify independent predictors of BRFS, DSS and MFS. Statistical significance was set at *p*< 0.05.

## 3. Results

### 3.1. Identification of the Top 10 Upregulated Genes Associated with Muscle Invasion and Distant Metastasis in the UBUC Transcriptome

**Figure 1:**
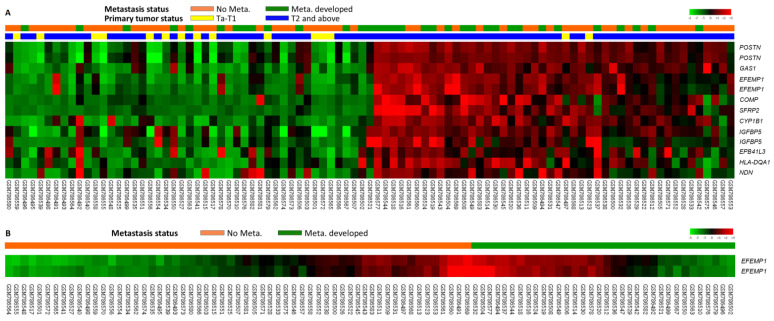
(**A**) Expression profiles of genes associated with the progression of urothelial carcinoma (muscle-invasive bladder cancer [MIBC] vs. non-muscle-invasive bladder cancer [NMIBC]; metastasis vs. non-metastasis) from a published transcriptome (GSE31684) in Gene Expression Omnibus. (**B**) *EFEMP1* is found to be one of the most significantly upregulated genes.

**Figure 2:**
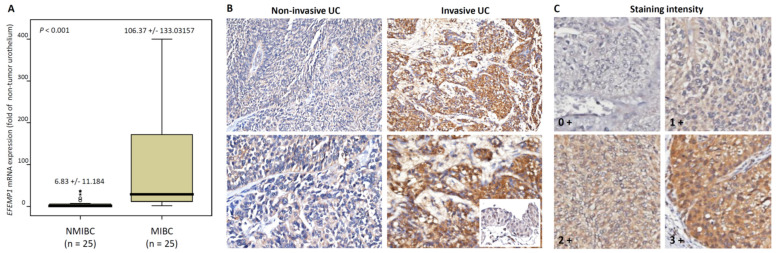
Expression of EFEMP1 mRNA and protein in urothelial carcinoma specimens. (**A**) *EFEMP1* mRNA level was significantly increased in MIBC (pT2-T4) using qRT-PCR. (**B**) Invasive UC showed high EFEMP1 expression using immunohistochemistry (normal urothelium in the inset) (upper: magnification × 200; lower: magnification × 400). (**C**) Immunostaing intensity. * *p* < 0.001.

We performed data mining of a GEO dataset (GSE31684), including 93 patients: 78 patients with MIBC and 34 with distant metastastic disease. Through transcriptomic profiling, we discovered the top 10 significantly upregulated genes associated with muscle invasion and distant metastasis in UBUC (Table 1 and Figure 1). *EFEMP1* was chosen for advance evaluation, because it was the most upregulated gene during the development of distant metastasis, which significantly affected UBUC patient outcomes. Furthermore, the oncologic functions of *EFEMP1* in UC are not well understood. We initially evaluated *EFEMP1* transcript expression in 50 snap frozen UBUC specimens. *EFEMP1* mRNA expression was significantly upregulated in patients with MIBC (*p* <0.001), suggesting its role in UC progression (Figure 2A).

These findings prompted us to further study the correlations between EFEMP1 protein levels and clinicopathological features and their prognostic roles in our large UTUC and UBUC cohorts.

### 3.2. Demographic Characteristics of our Cohort

We included 635 UC patients, including 340 UTUC and 295 UBUC (Table 2). There are 374 male and 261 female. In the UTUC group, 150 patients (44.1%) had ureteral UC and 62 patients (18.2%) had multifocal cancers. Moreover, 159 patients (46.8%) had advanced UTUC and 284 patients (83.5%) had high-grade tumors. Regarding lymph node status, 28 (8.2%) had lymph node metastatic UTUC at initial diagnosis. A total of 167 tumors (49.1%) had high mitosis, 106 (31.2%) had vascular invasion and 19(5.9%) had perineural invasion. In the UBUC group, 172 patients (58.3%) had NMIBC, 239(81%) had high-grade tumors and 29 (7.8%) had lymph node metastasis. Perineural invasion and vascular invasion were observed in 20 cases (6.8%) and 49 (16.6%), respectively. Furthermore,156 lesions (52.9%) showed high mitotic activity.

### 3.3. Correlations between EFEMP1Protein Levels and Important Clinicopathological Parameters

To confirm the relationship between EFEMP1 and UC, we used immunostaining to evaluate the EFEMP1 expression level (Figure 2B,C) and correlated its expression with clinicopathological features in UTUC and UBUC cohorts (Table 2). In the UTUC cohort, statistical analysis revealed that the EFEMP1expression level was significantly correlated with the primary pathologic T (*p* < 0.001), lymph node status (*p* < 0.001), histological grade (*p* = 0.001), vascular invasion (*p* < 0.001), mitotic rate (*p* = 0.039) and tumor location (*p* = 0.018). Similar results were observed in the UBUC cohort. High EFEMP1 immunoexpression was significantly associated with high primary pathologic T (*p* < 0.001), lymph node metastasis (*p* = 0.001), high histological grade (*p* < 0.001), vascular invasion (*p* < 0.001), perineural invasion (*p* = 0.021) and high mitosis (*p* < 0.001).

### 3.4. Prognostic and Survival Impacts of EFEMP1 Expression

The median follow-up period was 44.7 months for UTUCs and 30.8 months for UBUCs. There were 61 UTUC and 52 UBUC patient deaths due to UC progression. Moreover, 70 UTUC and 76 UBUC patients had subsequent tumor metastasis. We performed univariate and multivariate analyses to evaluate the survival significance of EFEMP1immunostaining level on patient death and cancer metastasis.

**Figure 3:**
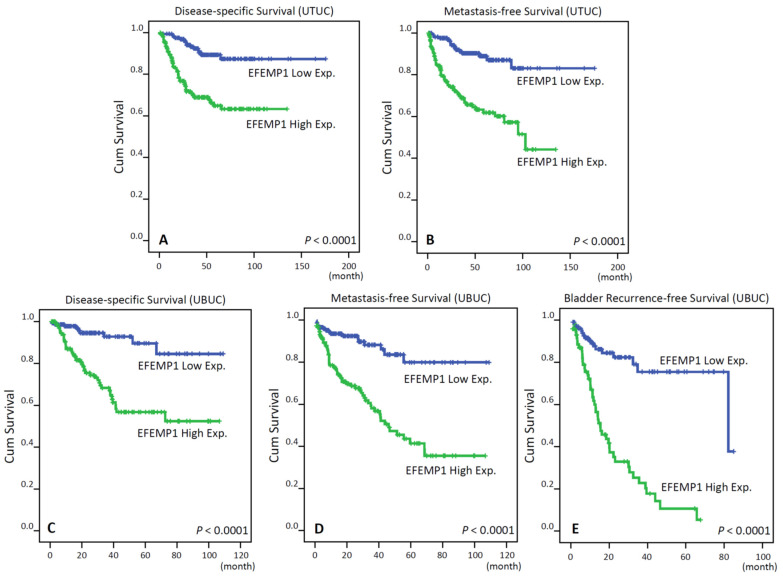
Kaplan-Meier plots show that EFEMP1 overexpression confers significant prognostic impacts in disease-specific survival, metastasis-free survival and bladder recurrence-free survival of patients with urothelial carcinoma of the upper tract (UTUC) (**A**,**B**, respectively) and urinary bladder (UBUC) (**C**–**E** respectively).

In UTUC (Table 3), high EFEMP1 expression levels contributed to higher rates of cancer-related deaths (27.1% vs. 8.8%) and postoperative cancer metastasis (31.2% vs.10.0%) than low EFEMP1 expression levels. Notably, in univariate analysis, high EFEMP1 immunoexpression (Figure 3A,B), high pT stage, metastastic lymph node, high tumor grade, vascular invasion, perineural invasion and multifocal tumors were significantly associated with worse DSS and MFS. Furthermore, multivariate Cox regression analysis revealed that EFEMP1 expression was an independent predictor of cancer-related death (*p* = 0.014; hazard ratio [HR], 2.233; 95% confidence interval [CI], 1.179–4.230) and metastasis occurrence (*p* =0.005; HR,1.21; 95% CI, 1.204–2.756).

In UBUC (Table 4), 43 patients (29.1%) with high EFEMP1-expressing tumors experienced cancer deaths and 60 patients (40.5%) had subsequent metastatic tumors, whereas only 16 patients (10.9%) with low EFEMP1-expressing tumors had caner metastasis and nine patients (6.2%) died of the disease. Notably, patients with high EFEMP1 expressing tumors had inferior DSS (Figure 3C; *p* < 0.0001) and MFS (Figure 3D; *p* < 0.0001) in the Kaplan-Meier survival analysis. In additiontoEFEMP1 immunostaining status, we found that pT stage, lymph node status, histological tumor grade, vascular invasion, perineural invasion and mitotic rate were associated with worse DSS and MFS in the univariate analysis. In the multivariate analysis, high EFEMP1immunoactivity (DSS: *p* < 0.001; HR, 4.181; 95% CI, 1.956–8.935; MFS: *p* < 0.001; HR, 3.163; 95% CI, 1.766–5.664) and high pathologic stage were markedly correlated with worse DSS and MFS. In the subgroup analysis of NMIBC, high EFEMP1-expressing NMIBCs correlated with a higher bladder tumor recurrence rate than low EFEMP1-expressing tumors (Figure 3E; *p* < 0.0001). Furthermore, adjusting tumor stage and grade, EFEMP1 expression status remained a significant prognostic factor for BRFS in multivariate analysis (Table 5).

### 3.5. Functional Enrichment Analysis of Dysregulated EFEMP1

We selected the top 200 most significant DEGs that were negatively or positively associated with *EFEMP1* expression in TCGA BLCA (Figures S1 and S2). The complete list and detailed information of these deregulated genes are presented in Tables S1 and S2. To determine the most significant canonical pathways and biological networks of *EFEMP1* involved in UC, we used IPA to examine the relationship between these highly significant genes; multiple canonical signaling pathways were enriched, including actin cytoskeleton signaling, tumor microenvironment pathway, mitochondrial dysfunction, ErbB2-ErbB3 signaling and ERK/MAPK signaling. The IPA analysis recognized *TGFB1*, *CCR2*, *HRAS*, *ACSS2* and *DGAT1* among the top upstream regulators.

Regarding disease and functions, we recognized that *EFEMP1* may be associated with cell movement, angiogenesis and cancers in the enrichment analyses. The top three most significant gene networks with scores > 42 were carbohydrate metabolism, small molecule biochemistry, vitamins and minerals, cell death and survival, cellular development, cellular function and maintenance, connective tissue development and function, organ morphology and tissue development.

## 4. Discussion

UTUC and UBUC are highly heterogeneous malignancies with varying biological and clinical behaviors. Patients with the same tumor stage may have different clinical outcomes. Identifying important molecular markers will assist physicians to establish personalized treatment strategies. Recently, we have discovered some biomarkers of UC, including TMCO1, SLC14A1 and MCM10 [20,21,22]. TMCO1, a novel tumor suppressor, dysregulated cell-cycle progression via suppression of the AKT pathway in UBUCs [20]. SLC14A1 prevented oncometabolite accumulation and inhibited the mTOR signaling pathway and subsequently UC tumorigenesis [21]. MCM10 overexpression implicated unfavorable clinicopathological characteristics and adverse prognosis in UC [22].

In this study, through transcriptomic data analysis, we found that *EFEMP1* was the most upregulated gene during the metastasis of UBUC. We then validated its prognostic role in our large cohort. Our results demonstrated that high EFEMP1 expression was associated with aggressive UC features. In NMIBC, high EFEMP1 immunoexpression was correlated with a high bladder tumor recurrence rate. Moreover, patients with high EFEMP1 expression increased the risks of UC-related cancer death and metastatic development in UTUC and UBUC.

Human *EFEMP1* is located on chromosome 2p16 [6,23]. It was first described to be overexpressed in senescent human fibroblasts established from a Werner syndrome patient, an inherited condition of premature aging [24]. A point mutation in *EFEMP1* causes an autosomal dominant macular degenerative disease caused by Malattia Levantine/Doyne honeycomb retinal dystrophy [25].Furthermore, genome-wide association studies have found that *EFEMP1* genetic variants, particularly rs3791679, are significantly associated with adult height [26], carpal tunnel syndrome [27] and inguinal hernia [28]. Notably, some of these conditions have also been found to involve increased EFEMP1 expression levels; therefore, this specific variant in the enhancer region may lead to pathological EFEMP1 overexpression. These findings suggest that EFEMP1 plays essential functions in regulating aging and maintaining the integrity of connective tissues.

As an important regulator in the extracellular matrix, including cell-to-cell and cell-to-matrix communication, EFEMP1 has been investigated in carcinogenesis [6,7].The deregulation of EFEMP1 in cancer development is complex [8,9,10,11,12,13,14,15,16,17,18]. It also has pro- and anti-tumorigenic activities, with up- or down-regulation of expression patterns depending on the cancer type. In breast cancer, *EFEMP1* is a new candidate tumor suppressor gene [14]. Sadr-Nabavi et al. demonstrated reduced EFEMP1 expression in breast cancer and its association with promoter methylation. Furthermore, low EFEMP1 expression correlated with poor clinical prognosis in patients with positive lymph node [11]. High EFEMP1 expression inhibits the progression of prostate cancer by suppressing cell proliferation and migration and promoting cell apoptosis [15]. In contrast, EFEMP1 was upregulated in osteosarcoma and significantly associated with worse survival and lymph node metastasis [9]. In glioma, increased EFEMP1expression promotes tumor invasion and progression by modulating the extracellular matrix by increasing the expression of MMP2, MMP9 and ADAMTS-5 via Notch signaling [8]. In pancreatic cancer, EFEMP1 binds to the EGF receptor and activates the Akt and MAPK pathways that enhance tumor growth [10]. However, the neoplastic roles of EFEMP1 in UTUC and UBUC have not been well studied.

In UBUC, TURBT with intravesical therapy is the standard treatment of NMIBC. High rates of tumor recurrence and progression are critical challenges in the clinical management of this disease [2]. A review of 19 trials showed that patients with NMIBC progression to MIBC had significantly decreased CSS than those with MIBC without a history of NMIBC [29]. In our study, EFEMP1 expression was higher in MIBC than in NMIBC and it can predict a high bladder recurrence rate after adjusting for tumor stage and grade, suggesting the prognostic role of EFEMP1 in NMIBC. EFEMP1 immunoexpression can help to identify patients with high-risk NMIBC that are most likely to benefit from aggressive treatment protocols. Furthermore, high EFEMP1 expression also predicted UBUC metastasis and cancer-related deaths. Integrated therapy using a radical cystectomy with perioperative chemotherapy may be beneficial for patients with high EFEMP1 expressing UBUC.

In UTUC, kidney-sparing surgery is suggested for low-risk cancers as patients’ survival is comparable to that of RNU and the surgical complications are decreased [1]. According to our results, high EFEMP1 expressing UTUC is associated with aggressive tumor features and a worse prognosis. Therefore, RNU should be considered in patients with low-risk UTUC but high EFEMP1 expression. Lymphadenectomy improves survival and local recurrence rate in patients with muscle-invasive UTUC (≥T2); however, tumor staging is inaccurate preoperatively [30]. We found that high EFEMP1 expression tumors were significantly correlated with muscle-invasive or lymph node metastatic UTUC. If high EFEMP1 expression is confirmed using biopsy specimens, RNU with lymph node dissection should be considered.

The biological and molecular roles of the EFEMP1 related pathways in UC are yet to be well elucidated. Some hypotheses have been proposed for other cancers. In osteosarcoma, EFEMP1 regulates cancer invasion and metastasis by inducing epithelial-mesenchymal transition and activating the NF-κB or Wnt/β-catenin signaling pathways [9,31]. In glioma, miR-338-5p targeting *EFEMP1* increases tumor apoptosis and suppresses tumor proliferation, migration and invasion [32]. EFEMP1 is also a novel autocrine/paracrine activator of Notch and NF-κB signaling. It enhances glioma invasion, growth, self-renewal, angiogenesis and resistance to apoptosis [8,33,34]. These clear pro-tumor properties highlight EFEMP1 as a putative therapeutic target. Nandhu et al. developed a function blocking antibody (mAb428.2) against EFEMP1. They confirmed anti-tumor efficacy against EFEMP1-secreting solid tumors (gliomas, lung cancers and kidney cancer) [35].

This study had some limitations. The first is the retrospective nature of our study. Second, the interpretation of EFEMP1 immunoexpression was not standardized. We evaluated its status using the H-score, which is highly correlated with Western blotting [15,16]. Third, the detailed molecular mechanisms by which EFEMP1promotes UC progression have not been studied. Using IPA, many important cancer-related pathways were enriched in UC, including the tumor microenvironment pathway, Rho family GTPases, integrin-linked kinase signaling and activated protein kinase signaling. Further investigation is needed to confirm the significance of these pathways in UC. Despite these limitations, the large well-characterized sample size, including UTUC and UBUC, is an important strength of our study, which increases the generalizability of our results.

## 5. Conclusions

Our data demonstrated that EFEMP1 expression was an independent prognostic factor for cancer death and metastasis in UTUC and UBUC. High EFEMP1 expression status is associated with tumor aggressiveness. Integrating EFEMP1 immunostaining with standard pathologic predictors can help urologists and their patients in clinical decision-making and risk stratification. This remains to be further elucidated and may be helpful as a therapeutic target. Elucidating the biological mechanisms of EFEMP1 in UC carcinogenesis may lead to a new strategy for effective treatment.

## Figures and Tables

**Table 1:** Summary of top 10 upregulated genes associated with muscle invasion and the development of distant metastasis in UC (GSE31684).

Probe	Comparing MIBC vs. NMIBC		Comparing Meta. vs. Non-Meta		Gene Symbol	Gene Title	Biological Process
	Log Ratio	*p*-Value	Log Ratio	*p*-Value			
201842_s_at	2.6528	<0.0001	1.6061	<0.0001	*EFEMP1*	EGF-containing fibulin-like extracellular matrix protein 1	visual perception
211959_at	2.6399	<0.0001	1.4678	<0.0001	*IGFBP5*	insulin-like growth factor binding protein 5	regulation of cell growth, signal transduction
212681_at	1.0972	0.0001	1.2658	<0.0001	*EPB41L3*	erythrocyte membrane protein band 4.1-like 3	cortical actin cytoskeleton organization and biogenesis
212671_s_at	1.2227	0.0078	1.2396	0.0004	*HLA-DQA1*	major histocompatibility complex; class II; DQ α 1, major histocompatibility complex; class II; DQ α 2, similar to HLA class II histocompatibility antigen; DQ(1) α chain precursor (DC-4 α chain)	antigen processing and presentation, antigen processing and presentation of peptide or polysaccharide antigen via MHC class II, immune response
201843_s_at	1.8283	<0.0001	1.2326	<0.0001	*EFEMP1*	EGF-containing fibulin-like extracellular matrix protein 1	visual perception
205713_s_at	1.8386	<0.0001	1.1823	0.0001	*COMP*	cartilage oligomeric matrix protein	cell adhesion, organ morphogenesis, skeletal development
202437_s_at	1.9076	<0.0001	1.1659	0.0007	*CYP1B1*	cytochrome P450; family 1; subfamily B; polypeptide 1	electron transport, visual perception
1555778_a_at	3.5717	<0.0001	1.1149	0.003	*POSTN*	periostin; osteoblast specific factor	cell adhesion, skeletal development
210809_s_at	3.8134	<0.0001	1.084	0.0079	*POSTN*	periostin; osteoblast specific factor	cell adhesion, skeletal development
203424_s_at	1.1435	0.0001	1.0825	<0.0001	*IGFBP5*	insulin-like growth factor binding protein 5	regulation of cell growth, signal transduction
204457_s_at	3.315	<0.0001	1.0793	0.005	*GAS1*	growth arrest-specific 1	cell cycle, cell cycle arrest, negative regulation of S phase of mitotic cell cycle, negative regulation of cell proliferation, programmed cell death
223121_s_at	2.1375	<0.0001	1.0374	0.0019	*SFRP2*	secreted frizzled-related protein 2	Wnt receptor signaling pathway, anterior/posterior pattern formation, cell differentiation, multicellular organismal development, somitogenesis
209550_at	0.7821	0.0044	1.0228	<0.0001	*NDN*	necdin homolog (mouse)	axon extension involved in development, axonal fasciculation, axonogenesis, central nervous system development, glial cell migration, negative regulation of cell proliferation, nerve growth factor receptor signaling pathway, nervous system development, neuron development, neuron migration, regulation of cell growth, regulation of progression through cell cycle, regulation of transcription; DNA-dependent, respiratory gaseous exchange, sensory perception of pain, transcription

**Table 2:** Correlations between EFEMP1 Expression and other important clinicopathological parameters in urothelial carcinomas.

Parameter	Category	Upper Urinary Tract Urothelial Carcinoma				Urinary Bladder Urothelial Carcinoma			
		Case No.	EFEMP1 Expression		*p*-Value	Case No.	EFEMP1 Expression		*p*-Value
			Low	High			Low	High	
Gender	Male	158	75	83	0.284	216	106	110	0.667
	Female	182	95	87		79	41	38	
Age (years)	<65	138	76	62	0.122	121	60	61	0.944
	≥65	202	94	108		174	87	97	
Tumor location	Renal pelvis	141	61	80	0.018 *	-	-	-	-
	Ureter	150	88	62		-	-	-	-
	Renal pelvis & ureter	49	21	28		-	-	-	-
Multifocality	Single	278	143	135	0.261	-	-	-	-
	Multifocal	62	27	35		-	-	-	-
Primary tumor (T)	Ta	89	70	19	<0.001 *	84	59	25	<0.001 *
	T1	92	54	38		88	41	47	
	T2-T4	159	46	113		123	47	76	
Nodal metastasis	Negative (N0)	312	166	146	<0.001 *	266	141	125	0.001*
	Positive (N1-N2)	28	4	24		29	6	23	
Histological grade	Low grade	56	39	17	0.001 *	56	41	15	<0.001 *
	High grade	284	131	153		239	106	133	
Vascular invasion	Absent	234	138	96	<0.001 *	246	134	112	<0.001 *
	Present	106	32	74		49	13	36	
Perineural invasion	Absent	321	164	157	0.098	275	142	133	0.021 *
	Present	19	6	13		20	5	15	
Mitotic rate (per 10 high power fields)	<10	173	96	77	0.039 *	139	92	47	<0.001 *
	≥10	167	74	93		156	55	101	

* Statistically significant.

**Table 3:** Univariate log-rank and multivariate analyses for disease-specific and metastasis-free survivals in upper urinary tract urothelial carcinoma.

Parameter	Category	Case No.	Disease-Specific Survival					Metastasis-Free Survival				
			Univariate Analysis		Multivariate Analysis			Univariate Analysis		Multivariate Analysis		
			No. of Event	*p*-Value	R.R.	95% C.I.	*p*-Value	No. of Event	*p*-Value	R.R.	95% C.I.	*p*-Value
Gender	Male	158	28	0.8286	-	-	-	32	0.7904	-	-	-
	Female	182	33		-	-	-	38		-	-	-
Age (years)	<65	138	26	0.9943	-	-	-	30	0.8470	-	-	-
	≥65	202	35		-	-	-	40		-	-	-
Tumor side	Right	177	34	0.7366	-	-	-	38	0.3074	-	-	-
	Left	154	26		-	-	-	32		-	-	-
	Bilateral	9	1		-	-	-	0		-	-	-
Tumor location	Renal pelvis	141	24	0.0079 *	1	-	0.817	31	0.0659	-	-	-
	Ureter	150	22		0.948	0.511–1.760		25		-	-	-
	Renal pelvis& ureter	49	15		1.461	0.406–5.258		14		-	-	-
Multifocality	Single	273	48	0.0026 *	1	-	0.217	52	0.0127 *	1	-	<0.001 *
	Multifocal	62	18		2.152	0.638–7.260		18		2.135	1.400–3.257	
Primary tumor (T)	Ta	89	2	<0.0001 *	1	-	0.234	4	<0.0001 *	1	-	0.487
	T1	92	9		3.293	0.702–15.446		15		1.286	0.714–2.317	
	T2-T4	159	50		3.784	0.816–17.543		51		0.946	0.487–1.836	
Nodal metastasis	Negative (N0)	312	42	<0.0001 *	1	-	<0.001 *	55	<0.0001 *	1	-	<0.001 *
	Positive (N1–N2)	28	19		5.223	2.818–9.678		15		3.064	1.827–5.139	
Histological grade	Low grade	56	4	0.0215 *	1	-	0.015 *	3	0.0027 *	1	-	0.057
	High grade	284	57		3.376	1.268–8.988		67		1.641	0.986–2.733	
Vascular invasion	Absent	234	24	<0.0001 *	1	-	0.200	26	<0.0001 *	1	-	0.066
	Present	106	37		1.491	0.809–2.747		44		1.559	0.971–2.502	
Perineural invasion	Absent	321	50	<0.0001 *	1	-	<0.001 *	61	<0.0001 *	1	-	<0.001 *
	Present	19	11		4.682	2.225–9.951		9		3.344	1.824–6.133	
Mitotic rate (per 10 high power fields)	<10	173	27	0.167	-	-		30	0.0823	-	-	
	≥10	167	34		-	-		40		-	-	
EFEMP1 expression	Low	170	15	<0.0001 *	1	-	0.014 *	17	<0.0001 *	1	-	0.005 *
	High	170	46		2.233	1.179–4.230		53		1.821	1.204–2.756	

* Statistically significant.

**Table 4:** Univariate log-rank and multivariate analyses for disease-specific and metastasis-free survivals in urinary bladder urothelial carcinoma.

Parameter	Category	Case No.	Disease-Specific Survival					Metastasis-Free Survival				
			Univariate Analysis		Multivariate Analysis			Univariate Analysis		Multivariate Analysis		
			No. of Event	*p*-Value	R.R.	95% C.I.	*p*-Value	No. of Event	*p*-Value	R.R.	95% C.I.	*p*-Value
Gender	Male	216	41	0.4446	-	-	-	60	0.2720	-	-	-
	Female	79	11		-	-	-	16		-	-	-
Age (years)	<65	121	17	0.1136	-	-	-	31	0.6875	-	-	-
	≥65	174	35		-	-	-	45		-	-	-
Primary tumor (T)	Ta	84	1	<0.0001 *	1	-	<0.001 *	4	<0.0001 *	1	-	<0.001 *
	T1	88	9		5.708	0.595–54.724		23		4.737	1.345–16.687	
	T2-T4	123	42		31.404	3.479–283.451		49		8.993	2.573–31.433	
Nodal metastasis	Negative (N0)	266	41	0.0002 *	1	-	0.912	61	<0.0001 *	1	-	0.167
	Positive (N1–N2)	29	11		1.041	0.509–2.129		15		1.557	0.831–2.915	
Histological grade	Low grade	56	2	0.0013 *	1	-	0.869	5	0.0007 *	1	-	0.694
	High grade	239	50		0.871	0.170–4.459		71		0.789	0.260–2.452	
Vascular invasion	Absent	246	37	0.0024 *	1	-	0.072	54	0.0001 *	1	-	0.846
	Present	49	15		0.530	0.266–1.059		22		0.941	0.510–1.738	
Perineural invasion	Absent	275	44	0.0001 *	1	-	0.082	66	0.0007 *	1	-	0.276
	Present	20	8		2.080	0.912–4.747		10		1.500	0.723–3.111	
Mitotic rate (per 10 high power fields)	<10	139	12	<0.0001 *	1	-	0.100	23	<0.0001 *	1	-	0.200
	≥10	156	40		1.796	0.894–3.636		53		1.420	0.831–2.426	
EFEMP1 expression	Low	147	9	<0.0001 *	1	-	<0.001 *	16	<0.0001 *	1	-	<0.001 *
	High	148	43		4.181	1.956–8.935		60		3.163	1.766–5.664	

* Statistically significant.

**Table 5:** Univariate log-rank and multivariate analyses for Bladder Recurrence-free Survivals in NMIBC post TURBT.

Parameter	Category	Case No.	Local Recurrence-Free Survival				
			Univariate Analysis		Multivariate Analysis		
			No. of Event	*p*-Value	R.R.	95% C.I.	*p*-Value
Gender	Male	125	46	0.3370	-	-	-
	Female	47	19		-	-	-
Age (years)	<65	70	30	0.3857	-	-	-
	≥65	102	35		-	-	-
Primary tumor (T)	Ta	84	27	0.0193 *	1	-	0.482
	T1	88	38		0.797	0.424–1.500	
Histological grade	Low grade	54	15	0.0101 *	1	-	0.139
	High grade	118	50		1.738	0.836–3.611	
Vascular invasion	Absent	171	65	0.6639	-	-	-
	Present	1	0		-	-	-
Perineural invasion	Absent	169	64	0.4725	-	-	-
	Present	3	1		-	-	-
Mitotic rate (per 10 high power fields)	<10	94	35	0.1853	-	-	-
	≥10	78	30		-	-	-
EFEMP1 expression	Low	100	17	<0.0001 *	1	-	<0.001 *
	High	72	48		5.502	3.037–9.968	

* Statistically significant.

## Data Availability

All data generated or analyzed during this study are included in this published article and its Supplementary Documentation File.

## Supplementary Materials

The following are available online at https://www.mdpi.com/article/10.3390/genes12060872/s1. Figure S1: The top 20 most significant differentially expressed genes that are positively correlated with EFEMP1 expression in bladder cancer. Figure S2: The top 20 most significant differentially expressed genes that are negatively correlated with EFEMP1 expression in bladder cancer. Table S1: The detailed information of positively deregulated genes. Table S2: The detailed information of negatively deregulated genes.
