# Supplementary figures and images for: Utility of EFEMP1 in the Prediction of Oncologic Outcomes of Urothelial Carcinoma
Source: Genes (Basel). 2021 Jun 6;12(6):872. doi: 10.3390/genes12060872 (PMC8226762; doi:10.3390/genes12060872)

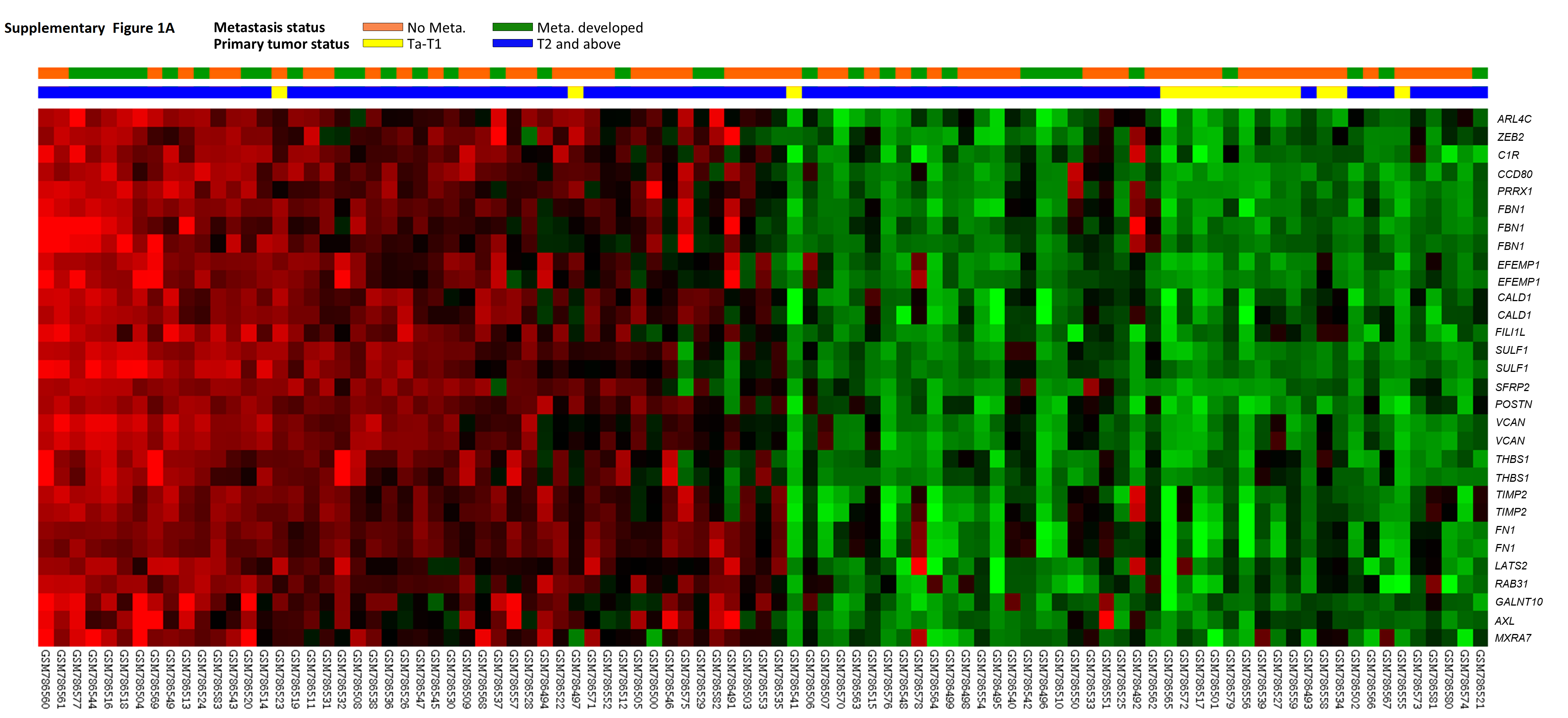

Supplement: Supplementary file 1 [file genes-12-00872-s001.zip › Figure S1.tif]

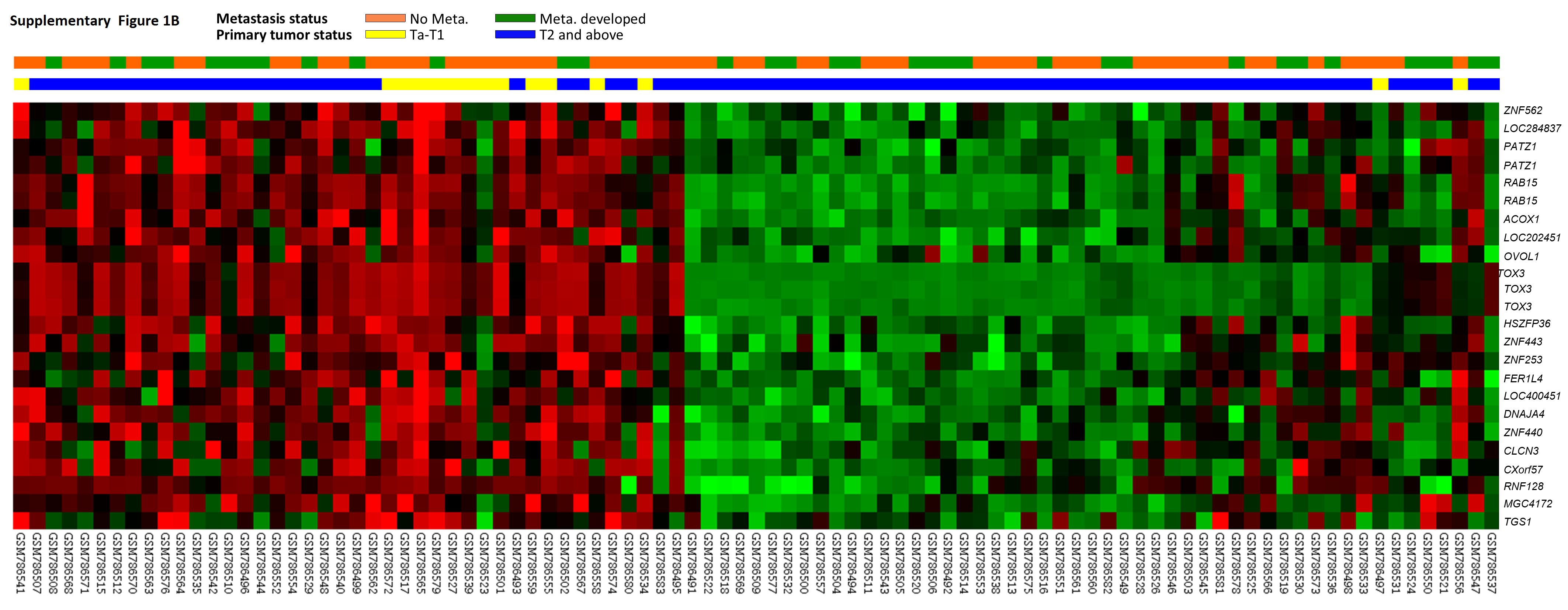

Supplement: Supplementary file 1 [file genes-12-00872-s001.zip › Figure S2.tif]
